# Fermentative Production of *N*-Alkylated Glycine Derivatives by Recombinant *Corynebacterium glutamicum* Using a Mutant of Imine Reductase DpkA From *Pseudomonas putida*

**DOI:** 10.3389/fbioe.2019.00232

**Published:** 2019-09-26

**Authors:** Melanie Mindt, Silvin Hannibal, Maria Heuser, Joe Max Risse, Keerthi Sasikumar, K. Madhavan Nampoothiri, Volker F. Wendisch

**Affiliations:** ^1^Genetics of Prokaryotes, Faculty of Biology and CeBiTec, Bielefeld University, Bielefeld, Germany; ^2^Fermentation Technology, Technical Faculty and CeBiTec, Bielefeld University, Bielefeld, Germany; ^3^Microbial Processes and Technology Division, National Institute for Interdisciplinary Science and Technology, Council of Scientific & Industrial Research, Trivandrum, India

**Keywords:** *Corynebacterium glutamicum*, imine reductase, metabolic engineering, enzyme engineering, *N*-alkylated amino acids, *N*-methylamino acids, *N*-ethylglycine, sarcosine

## Abstract

Sarcosine, an *N*-methylated amino acid, shows potential as antipsychotic, and serves as building block for peptide-based drugs, and acts as detergent when acetylated. *N*-methylated amino acids are mainly produced chemically or by biocatalysis, with either low yields or high costs for co-factor regeneration. *Corynebacterium glutamicum*, which is used for the industrial production of amino acids for decades, has recently been engineered for production of *N*-methyl-L-alanine and sarcosine. Heterologous expression of *dpkA* in a *C. glutamicum* strain engineered for glyoxylate overproduction enabled fermentative production of sarcosine from sugars and monomethylamine. Here, mutation of an amino acyl residue in the substrate binding site of DpkA (DpkA^F117L^) led to an increased specific activity for reductive alkylamination of glyoxylate using monomethylamine and monoethylamine as substrates. Introduction of DpkA^F117L^ into the production strain accelerated the production of sarcosine and a volumetric productivity of 0.16 g L^−1^ h^−1^ could be attained. Using monoethylamine as substrate, we demonstrated *N*-ethylglycine production with a volumetric productivity of 0.11 g L^−1^ h^−1^, which to the best of our knowledge is the first report of its fermentative production. Subsequently, the feasibility of using rice straw hydrolysate as alternative carbon source was tested and production of *N*-ethylglycine to a titer of 1.6 g L^−1^ after 60 h of fed-batch bioreactor cultivation could be attained.

## Introduction

*N*-methylated amino acids are non-proteinogenic amino acids found in plants, mammals, and microorganisms. Substitution of proteinogenic amino acids in a peptide with an *N*-methylated derivative mimics the native peptide, yet, often leads to increased stability against proteases and better membrane permeability (Chatterjee et al., 2013; Di Gioia et al., 2016). Further, *N*-methylated amino acids are known to stabilize—similar to l-proline—discrete conformations as shown for the ATPase inhibitor efrapeptin C (Dutt Konar et al., 2013). Natural peptides containing *N*-methylated amino acids such as the potential anti-cancer drugs enniatins, which disrupt membrane potential by forming stacked sandwich complexes with cations in the cell membrane (Kamyar et al., 2004), and bouvardin, which blocks protein synthesis by interacting with eukaryotic 80S ribosomes (Zalacaín et al., 1982), serve as a model for synthetic peptides (peptidomimetics). Several studies showed enhanced characteristics of peptidomimetics with incorporated *N*-methylated amino acids: for example Clingitide, a synthetic pentapeptide, showed increased affinity for integrin receptors (Mas-Moruno et al., 2010), while *N*-methyltubulysin showed enhanced antimitotic activity (Patterson et al., 2008). In addition to the incorporated amino acid derivatives, monomeric *N*-alkylated amino acids occur in nature as intermediates of metabolic pathways such as sarcosine (*N*-methylglycine) in degradation of creatine and choline, or *N*-methylglutamate in the C1 assimilation from monomethylamine by methylotrophic bacteria like *Methyloversatilis universalis* (Latypova et al., 2010). Free *N*-alkylated amino acids also occur in green tea leaves, e.g., the flavor enhancing *N*^5^-ethylated glutamine derivative l-theanine (Sakato, 1950). Studies on the effect of the anesthetic lidocaine showed pain reducing properties (Eipe et al., 2016). Further studies on this mechanism showed that the lidocaine metabolite *N*-ethylglycine (NEtGly) acts as an inhibitor of glycine uptake and inhibits pain signaling. Hence, NEtGly is a promising candidate for chronical pain treatment (Werdehausen et al., 2015).

Chemical synthesis of *N*-methylated amino acids has been studied extensively and main routes involve reductive amination, ring opening of 5-oxazolidinones and direct methylation (Aurelio et al., 2004; Gentilucci et al., 2010). Recently, the biocatalytic synthesis of *N*-alkylated amino acids has been developed (Hyslop et al., 2019). Notably, sustainable fermentative production of *N*-methylated amino acids was established in 2018 using two independent pathways. Pathway interception of the C1 assimilation pathway of *Methylobacterium extorquens* and implementation of the first two genes coding for γ-glutamylmethylamide synthetase and *N*-methylglutamate synthase enabled glycerol-based production of *N*-methylglutamate in *Pseudomonas putida* KT2440 (Mindt et al., 2018b). The second pathway for production of *N*-methylated amino acids exploited the side activity of the imine reductase DpkA from *Pseudomonas putida*. Natively, DpkA catalyzes the reduction of piperidein-2-carboxylate, but in addition DpkA catalyzes the reductive methylamination of 2-oxo acids. Expression of *dpkA* in recombinant *Corynebacterium glutamicum* strains overproducing the relevant 2-oxo acid precursor enabled fermentative production of *N*-methylalanine (NMeAla) (Mindt et al., 2018a) and sarcosine (Mindt et al., 2019), respectively, when monomethylamine (MMA) was added to the growth medium.

The industrially relevant production host *C. glutamicum* is used for decades for safe production of amino acids in the food and feed industries. Since the genetic tool box for *C. glutamicum* is developed very well, it was metabolically engineered for production of many value-added compounds, in particular proteinogenic amino acids, but also for non-proteinogenic amino acids like *trans*-hydroxyproline (Yi et al., 2014; Falcioni et al., 2015), pipecolic acid (Pérez-García et al., 2016, 2017a), 7-chloro- or 7-bromo-tryptophan (Veldmann et al., 2019a,b) and the *N*-methylated amino acids NMeAla (Mindt et al., 2018a) and sarcosine (Mindt et al., 2019). The immediate precursors of several amino acids, 2-oxo acids such as 2-oxovalerate (Krause et al., 2010), pyruvate (Wieschalka et al., 2012), and 2-oxoisocaproate (Bückle-Vallant et al., 2014) are also efficiently produced by recombinant *C. glutamicum* strains. The 2-oxo acid glyoxylate is not an immediate precursor of glycine biosynthesis in *C. glutamicum*. Nevertheless, a glyoxylate overproducing strain could be generated by interrupting the glyoxylate bypass of the TCA cycle (Zahoor et al., 2014) as basis for production of sarcosine (*N*-methylglycine) (Mindt et al., 2019).

By site-directed mutagenesis of the substrate binding site of the imine reductase DpkA, we could change the specific activity for methyl- and ethylamination of glyoxylate and this mutant led to faster fermentative production of sarcosine (*N*-methylglycine) and *N*-ethylglycine when expressed in the production strain. Potential of using biomass derived sugars for value added products such as *N*-ethylglycine was also demonstrated in a fed-batch bioreactor process.

## Materials and Methods

### Bacterial Strains and Culture Conditions

All bacterial strains, plasmids, and primers used were listed in Table 1. *Escherichia coli* DH5α (Hanahan, 1983) was cultivated in lysogeny broth (LB) at 37°C and 180 rpm. Pre-cultures of *C. glutamicum* were cultivated in LB, and main cultures for growth and production experiments where grown in standard CGXII medium (Eggeling and Bott, 2005) with reduced nitrogen content (2 g L^−1^ ammonium sulfate and 0.5 g L^−1^ urea) and given concentrations of carbon source and/or monomethylamine (MMA) at 30°C on a rotary shaker (120 rpm) in baffled shake flasks. All media were inoculated from a fresh LB or BHI agar plate, when necessary, ampicillin (100 μg mL^−1^), kanamycin (25 μg mL^−1^), spectinomycin (100 μg mL^−1^), and/or tetracycline (5 μg mL^−1^) were added to the medium. For induction of gene expression from the vectors pVWEx1, pEC-XT99A, and pEKEx3, medium was supplemented with 1 mm isopropyl-β-D-1-thiogalactopyranoside (IPTG). For growth and production experiments, *C. glutamicum* cells were harvested (3,200 × g, 7 min), washed once with TN buffer pH 6.3 (50 mm TrisHCl, 50 mm NaCl) and the minimal medium was inoculated to an optical density at 600 nm (OD_600_) of 1. Determination of OD_600_ was performed using V-1200 spectrophotometer (VWR, Radnor, PA, USA). Tolerance test of *C. glutamicum* for monoethylamine (MEA) was performed in the Biolector microfermentation system (m2p-labs, Aachen, Germany) in 48-well flower plates or in 100 mL flasks. Cultivation in 48-flower plates was performed in a volume of 1 mL at a shaking frequency of 1,200 rpm. Growth of cultures was followed by backlight scatter at 620 nm and at a signal gain factor of 20.

**Table 1:** Bacterial strains, vectors, and oligonucleotides used in this study.

**Strains, vectors, and oligonucleotides**	**Relevant characteristics**	**References**
***E. coli*** **strains**		
DH5α	*fhuA2 Δ(argF-lacZ)U169 phoA glnV44 Φ80 Δ(lacZ)M15 gyrA96 recA1 relA1 endA1 thi-1 hsdR17*	Hanahan, 1983
BL21(DE3)	*fhuA2 [lon] ompT gal (λ DE3) [dcm] ΔhsdS λ DE3 = λ sBamHIoΔEcoRI-B int::(lacI::PlacUV5::T7°gene1) i21 Δnin5*	Novagen
***C. glutamicum*** **strains**		
WT	*C. glutamicum* wild type, ATCC 13032	American Type Culture Collection
GLX	WT carrying deletion Δ*aceB* and the start codon exchange *icd^*GTG*^*	Zahoor et al., 2014
SAR3	WT carrying deletion Δ*aceB* and the start codon exchange *icd^*GTG*^* and vectors pVWEx1-*dpkA_*RBS^opt^ and pEC-XT99A-*xylA_*Xc*_-xylB_*Cg*_*	Mindt et al., 2019
SAR4	WT carrying deletion Δ*aceB* and the start codon exchange *icd^*GTG*^* and vectors pVWEx1-*dpkA_*RBS^opt^, pEC-XT99A-*xylA_*Xc*_-xylB_*Cg*_* and pEKEx3-*araBAD*	Mindt et al., 2019
SAR5	WT carrying deletion Δ*aceB* and the start codon exchange *icd^*GTG*^* and vectors pVWEx1-*dpkA*^F117L^ and pEC-XT99A-*xylA_*Xc*_-xylB_*Cg*_*	This work
SAR6	WT carrying deletion Δ*aceB* and the start codon exchange *icd^*GTG*^* and vectors pVWEx1-*dpkA*^F117L^, pEC-XT99A-*xylA_*Xc*_-xylB_*Cg*_*, and pEKEx3-*araBAD*	This work
**Plasmids**		
pET-22b	Amp^R^, production of N-terminal 10xHis-tagged proteins in *E. coli* (pBR322 oriV_E.c._, PT7, *lacI*)	Novagen
pET-22b-*dpkA*	Amp^R^, pET-22b expressing *dpkA* from *P. putida* KT2440 for protein purification	Mindt et al., 2019
pET-22b-*dpkA*^F117L^	Amp^R^, pET-22b expressing *dpkA*, carrying the mutation F117L from *P. putida* KT2440 for protein purification	This work
pVWEx1	Kan^R^, *C. glutamicum/E. coli* shuttle vector (P_tac_, *lacI*, pHM1519 oriV_C.g._)	Peters-Wendisch et al., 2001
pEKEx3	Spec^R^, *C. glutamicum/E. coli* shuttle vector (P_tac_, *lacI*, pBL1 OriV_C.g._)	Stansen et al., 2005
pEC-XT99A	Tet^R^, *C. glutamicum/E. coli* shuttle vector (P_trc_, *lacI*, pGA1 OriV_C.g._)	Kirchner and Tauch, 2003
pVWEx1-*dpkA*_RBS^opt^	Kan^R^, pVWEx1 expressing *dpkA* from *P. putida* KT2440 and change of start codon GTG to ATG and an optimized RBS	Mindt et al., 2019
pVWEx1-*dpkA*^F117L^	Kan^R^, pVWEx1 expressing *dpkA* from *P. putida* KT2440, carrying the mutation F117L, and change of start codon GTG to ATG and an optimized RBS	This work
pEC-XT99A-*xylA_*Xc*_-XylB_*Cg*_*	Tet^R^, pEC-XT99A expressing *xylA* from *Xanthomonas campestris* SCC1758, and *xylB* from *C. glutamicum* ATCC 13032	Veldmann et al., 2019a
pEKEx3-*araBAD*	Spec^R^, pEKEx3 expressing *araBAD* from *E. coli* MG1655	Pérez-García et al., 2017b
**Oligonucleotides**		
dpkA-pET-fw	GGCCATATCGAAGGTCGTCATATGTCCGCACCTTCCACCAG	Mindt et al., 2019
dpkA-pET-rv	CAGCCGGATCCTCGAGCATATCAGCCAAGCAGCTCTTTCAGG	Mindt et al., 2019
dpkA-pVW-fw	GCCAAGCTTGCATGCCTGCACAAGCGGCACAAATCGAGGTCGAAAAGGAGGTTTTTT TATGTCCGCACCTTCCACCAG	Mindt et al., 2019
dpkA-pVW-rv	GGGATCCTCTAGAGTCGACCTGCATCAGCCAAGCAGCTCTTTCA	Mindt et al., 2018a
dpkA_F117L_fw	GATCCACAACTCGCACCAT**CTG**GCTGCGTTGTG	This work
dpkA_F117L_rv	CACAACGCAGC**CAG**ATGGTGCGAGTTGTGGATC	This work

*Exchanged nucleotides for site-directed mutagenesis are depicted in bold*.

### Fed-Batch Bioreactor Process

For production of *N*-ethylglycine a stirred tank reactor cultivation of *C. glutamicum* was performed with an initial working volume of 2 L (3.7 L KLF, Bioengineering AG, Switzerland) at pH 7.0, 30°C, 0.2 bar overpressure, and an aeration rate of 1.25 NL min^−1^ enriched with 20% (v/v) CO_2_ in order to enhance carboxylating reactions in *C. glutamicum*. The pH value was controlled by the automatic addition of 10% (w/w) H_3_PO_4_ and 4 M KOH. Struktol® J647 serves as antifoam agent and was added manually when necessary. Two set-points for the relative dissolved oxygen saturation (rDOS) of 30% (stirrer speed) and 60% (feeding pump), respectively, serves for preventing first, oxygen limitations by enhancing stirrer speed gradually in steps of 2%, and second, substrate limitations by feeding of rice straw hydrolysate and potassium acetate (see section Preparation of Lignocellulosic Hydrolysates) until rDOS fell again under 60%. The initial stirrer speed was set to 200 min^−1^. Samples were taken automatically every 2 h within the first 34 h and every 4 h afterwards and kept to 4°C unil analysis. For fermentation, a BHI medium overnight culture of *C. glutamicum* strain SAR6 was used to inoculate a shake flask preculture with CGXII medium that contained reduced nitrogen content (2 g L^−1^ ammonium sulfate and 0.5 g L^−1^ urea) and rice straw hydrolysate as sole carbon source (at a concentration equivalent to 10 g L^−1^ sugars). The fermenter medium (2 L of CGXII minimal medium lacking MOPS buffer, containing reduced nitrogen content and rice straw hydrolysate as sole carbon source (at a concentration equivalent to 4 g L^−1^ sugars) was inoculated with 350 mL of this preculture. After reaching an optical density of about 5, 40 mL of 5 M monoethylamine solution were added. After 24 h of cultivation, 100 mL of 60 g L^−1^ potassium acetate solution were added.

### Preparation of Lignocellulosic Hydrolysates

Rice straw procured from a local market in Trivandrum, Kerala (India), was used as the raw material for the hydrolysis. Dried rice straw was powdered in a knife mill to a particle size ≤1 mm and sealed in polythene bags till further use. The milled and processed biomass was treated using diluted acid (H_2_SO_4_) with slight modifications on the method described by Gopinath et al. (2011). A solid loading of 10% (dry w/v) and an acid loading of 4% (v/v) were used for the hydrolysis at 134°C for 35 min. The hydrolysed biomass obtained was cooled, neutralized with 10 m KOH and wet sieved. The liquid fraction was centrifuged at 4,000 rpm for 20 min to remove remaining particles and filter-sterilized (Millipore, Massachusetts, USA) before fermentation.

### Molecular Genetic Techniques and Strain Construction

Transformation of competent *Escherichia coli* DH5α (prepared by RbCl method) was performed by heat shock (Green and Sambrook, 2012) and transformation of *C. glutamicum* by electroporation (Eggeling and Bott, 2005). PCR-fragments were amplified using the respective primer (see Table 1) with ALLin™ HiFi DNA Polymerase (highQu GmbH, Kraichtal, Germany). The mutation F117L was introduced into *dpkA* using the respective primers (see Table 1) followed by cross-over PCR for alignment of the PCR products. PCR fragments were assembled to linearized vectors (pET-22b with NdeI, pVWEx1 with BamHI) by Gibson assembly (Gibson et al., 2009). The expression host *E. coli* BL21(DE3) was transformed with plasmids for protein purification (pET-22b), *C. glutamicum* was transformed with expression vectors pVWEx1, pEKEx3, and pEC-XT99A.

### Enzyme Preparation and Kinetic Characterization

For enzyme characterization, fresh cultures of *E. coli* BL21(DE3) derived strains were inoculated from an overnight culture to an OD_600_ of 0.05 in 500 mL LB in 2 L baffled flask. When cultures reached OD_600_ of 0.5–0.6, 1 mm IPTG was added for induction of gene expression and cultures were transferred to 20°C and 180 rpm for further 3.5 h. Subsequently, cells were harvested at 4°C and cell pellets were stored at −20°C for further analysis. Starting with crude extract preparation, cells, and enzyme solutions were kept on ice or at 4°C. Crude extract preparations, protein purification (Mindt et al., 2019), and reductive activity assays (Muramatsu et al., 2005; Mindt et al., 2018a) were performed as described previously. For determination of Michaelis constants (K_m_), various 2-oxo acid concentrations from 0.5 to 30 mm were added. The K_m_ values were determined using Origin with the add-on “Enzyme kinetics” and calculations for k_cat_ were performed with respect to Michaelis and Menten (translation: Johnson and Goody, 2011) (M_DpkA_ = 35.14 kDa). Specific activity was given in units, where one unit (U) was defined as the amount of enzyme required to convert 1 μmol substrate within 1 min.

### Quantification of Amines, Organic Acids, and Sugars in Fermentation Broth

Amines, oxo acids, and carbohydrates were determined using high-performance liquid chromatography (HPLC) (1200 series, Agilent Technologies Deutschland GmbH, Böblingen, Germany). For quantification, supernatants of the cultures were harvested regularly by centrifugation (20,200 × g, 15 min at 4°C) and stored at −20°C for further analysis. Derivatization of amines with 9-fluorenylmethyl chlorocarbonate was performed as described elsewhere (Mindt et al., 2018b). Separation was performed on a reversed phase HPLC using a column system consisting of a pre-column (LiChrospher 100 RP18 EC-5μ (40 × 4 mm), CS-Chromatographie Service GmbH, Langerwehe, Germany) and a main column (LiChrospher 100 RP18 EC-5μ (125 × 4 mm), CS Chromatographie Service GmbH). Fluorescent derivatives were detected by a fluorescence detector (FLD G1321A, 1200 series, Agilent Technologies) with excitation and emission wavelength of 263 and 310 nm, respectively. l-proline was used as internal standard.

Concentrations of organic acids and sugars were determined using an amino exchange column (Aminex, 300 × 8 mm, 10 μm particle size, 25 Å pore diameter, CS Chromatographie Service GmbH) with 5 mm sulfuric acid as mobile phase and a refractive index detector (RID G1362A, 1200 series, Agilent Technologies) at 210 nm.

## Results and Discussion

### Site-Directed Mutagenesis of the Active Site of the *N*-Methylamino Acid Dehydrogenase DpkA

In a previous study, we established fermentative production of sarcosine by methylamination of glyoxylate using DpkA and showed that xylose was superior as source of carbon and energy than glucose (Mindt et al., 2019). To improve the low volumetric productivities observed for this sarcosine production process, we focused on changing an amino acyl residue of the binding site of the enzyme DpkA here.

**Figure 1:**
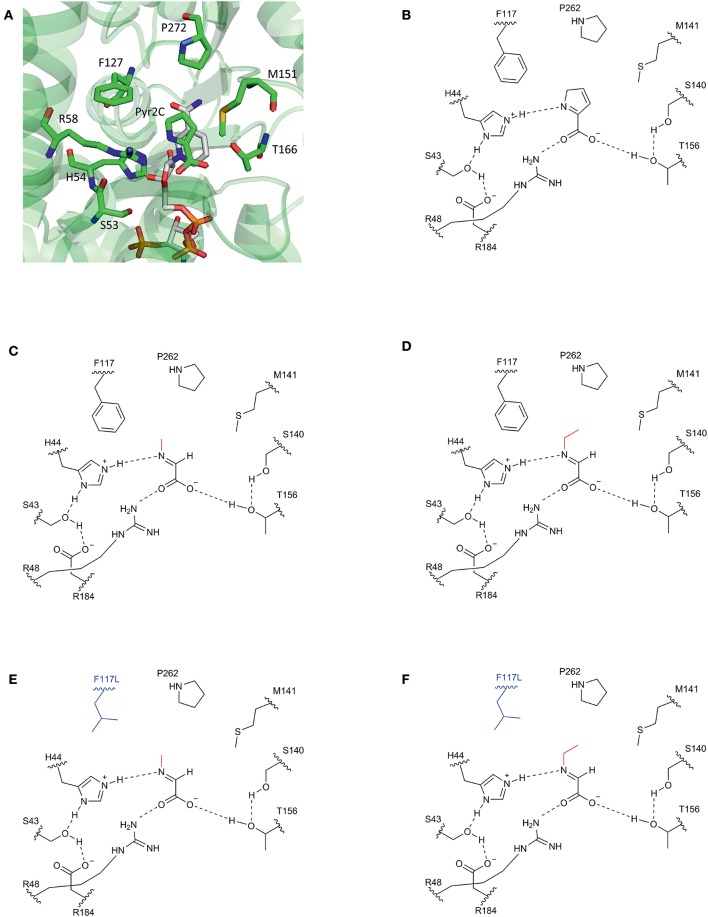
Schematic view of the substrate binding site of DpkA. **(A)** Active site of DpkA from *Pseudomonas syringae* (PDB: 2CWH). The native substrate pyrroline-2-carboxylate (Pyr2C; carbon atoms in green) and the cofactor NADPH (carbon atoms in light gray) are bound to the active site. The pyrrole ring of Pyr2C is recognized by the three amino acid residues Phe117, Pro262, and Met141 (carbon atoms in green). Schematic view of the active site of DpkA from *Pseudomonas putida* with the native substrate Pyr2C **(B)**, the imine formed by glyoxylate and MMA **(C)**, and the imine formed by glyoxylate and MEA **(D)**. Potential substrate binding site of the mutant DpkA^F117L^ with the imine formed by glyoxylate and MMA **(E)** and the imine formed by glyoxylate and MEA **(F)**. The alkyl moiety from the alkylamine is shown in red. The mutated moiety Phe117Leu is depicted in blue (Based on Goto et al., 2005).

Our approach was based on the protein structure of a DpkA homolog from *Pseudomonas syringae* (PsDpkA) for which the residues of the active site could be identified by co-crystallization of PsDpkA with the native substrate pyrroline-2-carboxylate (Pyr2C) and redox cofactor NADPH (Figure 1; PDB: 2CWH; Goto et al., 2005). The catalytic site comprises S43, H54, R58, and T166 and substrate binding involves the three amino acid residues F127, M151, and P272 (Figure 1A; Goto et al., 2005). These residues are conserved in DpkA from *P. putida* KT2440 that shares amino acid similarity of 79.6% (catalytic site: S33, H44, R48, and T156; substrate binding site: F117, M141, and P262; Figure 1B). Due to structural similarities of the native substrate Pyr2C and the imines formed by 2-oxo acids and alkylamines, we assumed that the non-native imine substrates are recognized in a similar manner. The wild-type DpkA and several mutants (Table 2) were purified from recombinant *E. coli* and their specific activity for the reductive amination of glyoxylate using MMA was determined. Mutagenesis of the three residues F117, M141, and P262 revealed altered specific activities for the reductive amination of glyoxylate using MMA (Table 2). The mutations P262I, P262L (data not shown), and P262G led to almost complete loss of activity, while the mutation P262A reduced the activity to 30% of the wildtype enzyme. Combined mutagenesis of positions P262 and M141 showed only minor improvement of the specific reductive amination of glyoxylate. Additionally, mutation F117L was considered under the assumption that it favors aliphatic interaction with imines formed by glyoxylate and either MMA or MEA (Figure 1). Although being aware that residues are often changed to Ala, our intention was to keep the aliphatic character of the binding pocket due to potential interactions with the alkyl-moiety of the *N*-alkylated product. We did not choose Ala since alanine is much smaller compared to phenylalanine (in the wildtype, which showed high activity for methylation) and our aim was to increase the activity toward ethylation. Since the size of the desired product increased by one carbon (from methylation to ethylation), we chose the aliphatic leucine as substitution to increase the binding pocked slightly and intended a potential aliphatic interaction of the leucine residue and the *N*-alkyl moiety. This mutant (DpkA^F117L^) was the only one in the chosen library which showed improved activity for the reductive amination of glyoxylate.

**Table 2:** Specific activity of different 10xHis-DpkA mutants with glyoxylate and MMA as substrates.

**Enzyme**	**Specific activity (U mg^**−1**^)**
DpkA	25.7 ± 1.8
DpkA^F117L^	30.3 ± 2.7
DpkA^P262G^	0.6 ± 0.1
DpkA^P262A^	8.4 ± 2.7
DpkA^P262AM141V^	11.6 ± 0.5
DpkA^P262AM141L^	1.9 ± 0.3

*The enzymes were purified from E. coli BL21 (DE3) using nickel chelate chromatography. The specific activity was determined in a volume of 1 mL containing 100 mM glycine buffer (pH 10), 60 mM MMA, 10 mM glyoxylate, and 0.3 mM NADPH. The consumption of NADPH was detected at 340 nm at 30°C for 3 min*.

**Figure 2:**
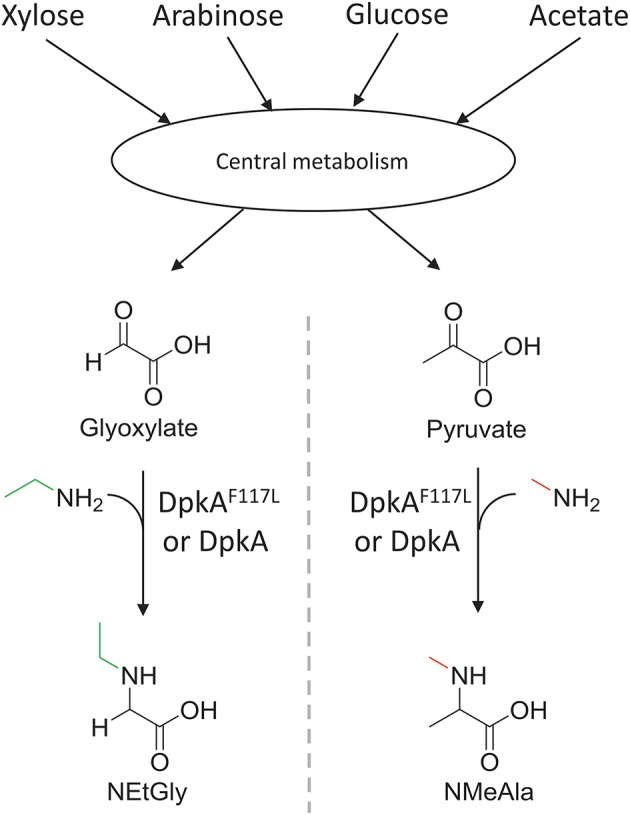
Schematic view of the sugar-based production of *N*-alkylated amino acids by *C. glutamicum*. Sugars (C5 and C6) and acetate of lignocellulosic hydrolysates are catabolized in the central carbon metabolism and converted to 2-oxo acids. Subsequently, the imine reductase DpkA from *P. putida* catalyzes the reductive amination of the 2-oxoacids yielding *N*-alkylated amino acids. The mutant DpkA^F117L^ catalyzes the faster formation of NEtGly than the wild-type enzyme.

The mutant DpkA^F117L^ was the most promising one in the prior analysis, therefore the substrate affinities (pyruvate and glyoxylate as 2-oxo acid substrates, MMA and MEA as alkylamine substrates) and catalytic efficiencies of wildtype DpkA and DpkA^F117L^ were determined (see Supplementary Figures S1, S2). Interestingly, characterization of the wild-type enzyme DpkA with non-native substrates revealed substrate preference for glyoxylate and MEA over glyoxylate and MMA. Methylamination of pyruvate showed the highest catalytic efficiency (Table 3). Mutation of phenylalanine at position 117 by leucine enabled an increased specific methylamination activity toward glyoxylate (30.3 ± 2.7 U mg^−1^; wild-type enzyme 25.7 ± 1.8 U mg^−1^; Mindt et al., 2019), while the catalytic efficiency was similar. Yet, the catalytic efficiency for reductive methylamination of pyruvate (7.95 s^−1^ mm^−1^, wt: 6.66 s^−1^ mm^−1^) and for reductive ethylamination of glyoxylate (7.61 s^−1^ mm^−1^, wt: 6.44 s^−1^ mm^−1^) was enhanced by this mutant (Table 3; Figure 2). Further biochemical characterization of the mutant DpkA^F117L^ may unravel the influence of this phenylalanine residue on substrate binding of other non-native aliphatic substrates as well as of the native substrates (Pip2C and Pyr2C). Thus, the mutant DpkA^F117L^ is the most promising candidate to enhance volumetric productivity for fermentative production of sarcosine as well as NEtGly. While a thorough biochemical analysis of DpkA and its mutants is required to unravel the underlying molecular mechanism, our aim to improve reductive ethylamination of glyoxylate was met.

**Table 3:** Parameters of 10xHis-DpkA and the mutant 10xHis-DpkA^F117L^ with various 2-oxo acid substrates and MMA and MEA as amine substrates.

**Enzyme**	**Amine substrate**	**2-Oxo acid substrate**	**K_m_ (mM)**	**sp. act. (U mg^**−1**^)**	**k_**cat**_ (s^**−1**^)**	**Catalytic efficiency (s^−1^ mm^−1^)**
DpkA	MMA	Pyr	3.3 ± 0.5	37.5 ± 1.7^*^	22.0	6.66
	MEA	Pyr	10.6 ± 0.5	2.0 ± 0.2^*^	1.2	0.15
	MMA	Glx	5.4 ± 0.8	25.7 ± 1.8	15.1	2.79
	MEA	Glx	2.3 ± 0.3	25.3 ± 3.2	14.8	6.44
DpkA^F117L^	MMA	Pyr	2.3 ± 0.6	31.2 ± 1.3	18.3	7.95
	MEA	Pyr	10.7 ± 2.2	1.9 ± 0.2	1.1	0.10
	MMA	Glx	6.7 ± 1.6	30.3 ± 2.7	17.3	2.55
	MEA	Glx	2.4 ± 0.7	31.2 ± 1.1	18.3	7.61

* *Specific activities have been determined in Mindt et al. (2019)*.

*The enzymes were purified from E. coli BL21 (DE3) using nickel chelate chromatography. The specific activity was determined in a volume of 1 mL containing 100 mM glycine buffer (pH 10), 60 mM MMA or 200 mM MEA, 10 mM 2-oxoacid, and 0.3 mM NADPH. The consumption of NADPH was detected at 340 nm at 30°C for 3 min. For K_m_ value determination, different concentrations of 2-oxoacids were added (0.5–30 mM) with 60 mM MMA or 200 mM MEA. Means and standard deviations of triplicates are given. Pyr, pyruvate; Glx, glyoxylate. The Michaelis-Menten kinetics are shown in the supplements (see Supplementary Figures S1, S2)*.

The kinetic studies of Mihara et al. on DpkA revealed a sequential Ter Bi mechanism: NADPH, pyruvate, and methylamine sequentially bind to the active site, followed by sequential release of NMeAla and NADP^+^ (Mihara et al., 2005). Due to this finding, DpkA may catalyze imine formation in addition to the reduction of the imine to its corresponding amine. Additional to catalysis by *N*-methylamino acid dehydrogenases, imine formation is catalyzed by other enzyme classes like amino acid dehydrogenases (Brunhuber et al., 2000) and reductive aminases (Aleku et al., 2017; Sharma et al., 2018). Surprisingly, several studies of classical imine reductases demonstrated that IREDs catalyze imine reduction, but not their formation from (alkyl)amines and ketones (Huber et al., 2014; Scheller et al., 2015; Wetzl et al., 2016). For the reductive alkylamination of 2-oxo acids by DpkA via a sequential Ter Bi mechanism, the presence of different alkylamines should not change K_m_ values for the same 2-oxo acid since the 2-oxo acid is expected to bind prior to the alkylamine. However, the substrate affinity of the wild type enzyme for glyoxylate in the presence of MMA (K_m_ 5.4 mm) was more than two times lower than in the presence of MEA (K_m_ 2.3 mm; Table 3). Due to the contradictory findings to the study of Mihara et al. (2005), further investigation of the reaction mechanism for different non-native substrate combinations and a detailed analysis of the coordination of non-native substrates in the active site are needed to rationally engineer DpkA. Depending on the knowledge of the structure and the reaction mechanism, there are three different approaches for protein engineering: random, rational and semi-rational (Arnold, 2001; Reetz and Carballeira, 2007; Gupta and Tawfik, 2008). Alternatively, semi-rational enzyme design, in which the structural information is taken into account to generate mutant libraries (Lutz, 2010), may be used. This strategy was successfully applied e.g., to change an leucine dehydrogenase from *Bacillus stereothermophilus* to an enantioselective amine dehydrogenase (Abrahamson et al., 2012) or to change the cofactor specificity of an IRED from NADPH to NADH (Borlinghaus and Nestl, 2018).

### Expression of DpkA^F117L^ in *C. glutamicum* Led to Enhanced Sarcosine and *N*-Ethylglycine Production

**Figure 3:**
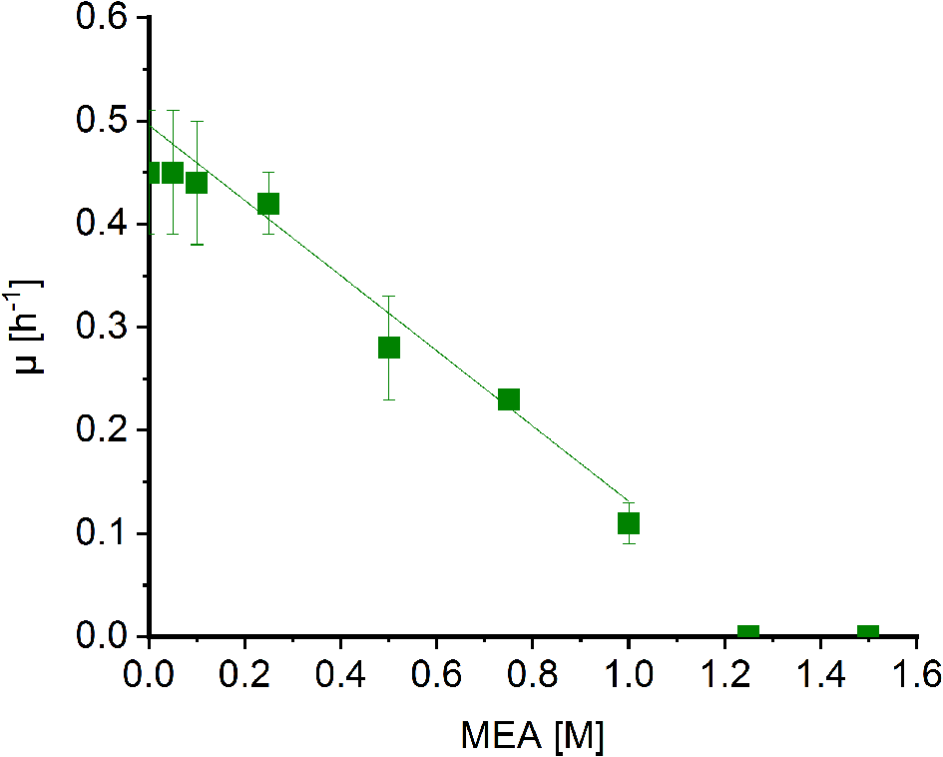
Effect of MEA on growth of *Corynebacterium glutamicum* wild type. Growth rates of *C. glutamicum* cultivated in minimal medium supplemented with increasing concentrations of MEA (green boxes) in Biolector microfermentation system in 48-well flower plates. Effect of MEA for half maximal growth rates was determined by extrapolation and linear fitting (OriginLab, Northampton, MA).

*Corynebacterium glutamicum* was shown to withstand high concentrations of MMA (Mindt et al., 2018a) and sarcosine (Mindt et al., 2019). In order to determine possible effects of MEA on growth of *C. glutamicum*, cells were grown in minimal medium supplemented with increasing concentrations of MEA (0.05–1.5 m). Extrapolation of the graph revealed a diminished growth rate to about half maximal at MEA concentrations of 0.7 m (Figure 3). Albeit the C2-compound showed a stronger negative impact on growth of *C. glutamicum* compared to methylamine (1.8 m; Mindt et al., 2018a), high concentrations of both alkylamines are tolerated by *C. glutamicum*, and hence, this bacterium is a suitable host for fermentative production of *N*-alkylated amino acids.

**Figure 4:**
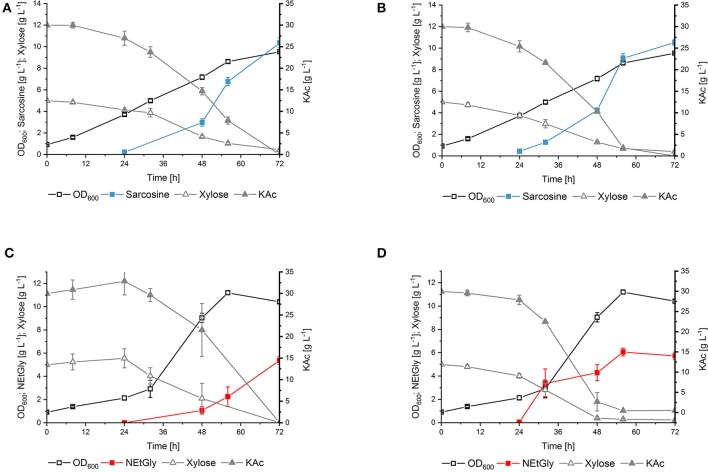
Production sarcosine and NEtGly by *C. glutamicum* strains in shake flask fermentation. The growth, production, and consumption of carbon source of SAR3 **(A,C)** and SAR5 **(B,D)** in minimal medium supplemented with 5 g L^−1^ xylose, 30 g L^−1^ potassium acetate, and 6.2 g L^−1^ MMA **(A,B)** or 9.0 g L^−1^ MEA **(C,D)** was followed. Biomass formation (open black boxes), sarcosine production (filled turquoise boxes), NEtGly production (filled red boxes), xylose (open gray triangles), and potassium acetate (filled gray triangles) are given as means of triplicates with standard deviations.

To compare the influence of the substitution of phenylalanine residue at position 117 to leucine in DpkA on sarcosine production, vector pVWEx1-*dpkA*^F117L^ was constructed. This vector differs from vector pVWEx1-*dpkA*_RBS^°pt^ (Mindt et al., 2019) only by the amino acid exchange F117L. Transformation of the glyoxylate production strain with this plasmid yielded strain SAR5 [i.e., strain GLX(pVWEx1-*dpkA*^F117L^) (pEC-XT99A-*xylA*_Xc_-*xylB*_Cg_); Table 1]. Strain SAR5 and isogenic strain SAR3 [i.e., strain GLX(pVWEx1-*dpkA*_RBS^°pt^) (pEC-XT99A-*xylA*_Xc_-*xylB*_Cg_); Table 1] were cultivated in minimal medium supplemented with 5 g L^−1^ xylose, 30 g L^−1^ potassium acetate and 6.2 g L^−1^ MMA (i.e., 200 mm). Production of sarcosine from both strains started 24 h after inoculation and yielded similar titers after 72 h cultivation (SAR3 10.4 ± 0.5 g L^−1^; SAR5 10.5 ± 0.3 g L^−1^). Notably, the mutated imine reductase DpkA^F117L^ allowed faster fermentative production of sarcosine (vol. prod. for 56 h: 0.16 g L^−1^ h^−1^) compared to the wild type enzyme (vol. prod. for 56 h: 0.12 g L^−1^ h^−1^) (Figures 4A,B). Carbon source utilization by SAR5 was faster than by SAR3. SAR5 stopped NEtGly production earlier (at around 48 h), which we believe is due to the fact that the carbon sources were depleted. Lactate was not detected as byproduct. Although DpkA and DpkA^F117L^ showed higher catalytic efficiency for methylamination of pyruvate as compared to glyoxylate, the finding that neither strain produced *N*-methylalanine as side product indicates that the glyoxylate overproduction base strain provided sufficient glyoxylate while maintaining sub-threshold pyruvate concentrations.

DpkA favors reductive ethylamination of glyoxylate over reductive ethylamination of pyruvate by one order of magnitude (6.44 s^−1^ mm^−1^ as compared to 0.15 s^−1^ mm^−1^; Table 3). This preference is even larger for the mutant DpkA^F117L^ (7.61 s^−1^ mm^−1^as compared to 0.10 s^−1^ mm^−1^; Table 3). Since the K_m_ values for glyoxylate were in the lower millimolar range for DpkA (around 2–7 mM; Table 3), we expected that reductive ethylamination of glyoxylate should proceed *in vivo*. Thus, both strains expressing the mutant or the wild type enzyme (SAR5 and SAR3, respectively) were cultivated with 9.0 g L^−1^ MEA (or 200 mm) instead of MMA. Indeed, both strains produced NEtGly to comparable product titers (5.7 ± 0.3 g L^−1^ for SAR5 and 5.4 ± 0.3 g L^−1^for SAR3 accumulated within 72 h; Figures 4C,D), while NEtAla was not formed as by-product. As expected from the higher catalytic efficiency of DpkA^F117L^, strain SAR5 showed faster consumption of potassium acetate and xylose as well as faster NEtGly production (volumetric productivity within 56 h: SAR5 0.11 g L^−1^ h^−1^, SAR3 0.04 g L^−1^ h^−1^). To the best of our knowledge, this is the first demonstration of fermentative production of NEtGly.

Interestingly, the strains showed different growth behavior during production of sarcosine und NEtGly. Growth of the engineered *C. glutamicum* strains (both SAR3 and SAR5) in NEtGly producing experiments is improved compared to sarcosine production (Figure 4). This may be due to the lower k_cat_ value of DpkA and DpkA^F117L^ for the reductive amination of glyoxylate with MEA (Table 3). Accumulation of glyoxylate intracellularly inhibits the growth of *C. glutamicum* (Zahoor et al., 2014), thus, faster consumption of the aldehyde in presence of MEA may allow for faster growth.

### Production of NEtGly From Lignocellulosic Hydrolysates

**Figure 5:**
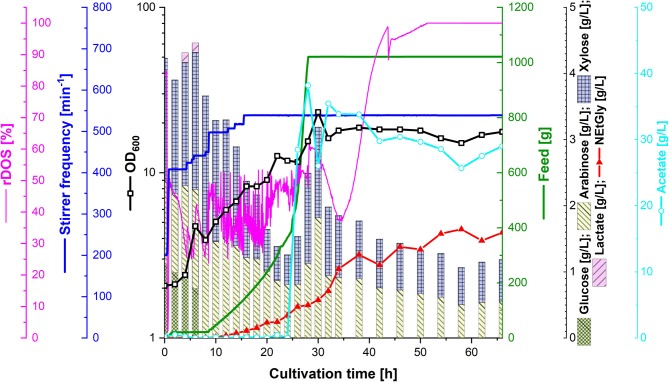
Production of NEtGly from lignocellulosic hydrolysate by *C. glutamicum* SAR6 in a 2 L scale fed-batch bioreactor process. The fermentation took place in low nitrogen minimal medium supplemented with feed (green line) consisting of rice straw hydrolysate and 18 g L^−1^ potassium acetate (1:1.85 ratio). After cells reached an OD_600_ (open black boxes) of 5, 40 mL of 5 M ethylamine solution were added; after 24 h of cultivation, 100 mL of 60 g L^−1^ potassium acetate solution were added. The relative dissolved oxygen saturation (rDOS; pink line) was measured to control the stirrer frequency (dark blue line) and the feed supplementation as described in section Fed-Batch Bioreactor Process. Samples for HPLC analysis of sugar content (cumulative stacked columns), acetate content (light blue open circles) and NEtGly production (red triangles) were taken automatically.

In order to test the efficacy of lignocellulosic hydrolysate for fermentative production of NEtGly, strain SAR5 was transformed with plasmid pEKEx3-*araBAD* to yield strain SAR6, which is able to also consume arabinose. The hydrolysis of rice straw with 4% sulfuric acid (see section Preparation of Lignocellulosic Hydrolysates) resulted in 14.7 g L^−1^ of xylose, 7.2 g L^−1^ of glucose, 4.3 g L^−1^ of arabinose, and 4.3 g L^−1^ of acetate. Strains SAR4 and SAR6 are able to utilize the four carbon sources xylose, glucose, arabinose, and acetate present in the hydrolysis liquor. The hydrolysis liquor was mixed with 18 g L^−1^ potassium acetate (1:1.85 ratio) to obtain the feed used during fermentation. During the first growth phase of the fermentation process (up to 8 h of cultivation, see Figure 5), glucose was depleted completely and lactate accumulated transiently up to a concentration of 0.1 g L^−1^. At that time, i.e., after reaching an optical density of about 5, 40 mL of 5 M ethylamine solution was added. From 8 to 24 h of cultivation, the feed was increased. While xylose and arabinose concentrations in the bioreactor medium varied between 0.5 and 2 g L^−1^, glucose was not detected and only little acetate was observed. During this period, growth continued and NEtGly production started. After 24 h of cultivation, 100 mL of 60 g L^−1^ potassium acetate solution were added and after 28 h the feed was stopped. After 30 h of cultivation, growth reached the maximal OD_600_ of 23.3 (Figure 5). In the last phase (from 30 to 66 h), acetate and the pentoses were utilized slowly. NEtGly was produced steadily and the concentration more than doubled from 30 to 66 h. A final concentration of 1.6 g L^−1^ of NEtGly was obtained.

Bioreactor cultivation showed that the production of NEtGly is scalable and can be based on lignocellulosic hydrolysate. However, titer, yield, and productivity were lower than in shake flasks experiments using pure chemicals as carbon sources. To some extent, *C. glutamicum* shows recalcitrance against typical inhibitors present in lignocellulosic hydrolysates such as hydroxymethylfurfural and methylfurfural (Gopinath et al., 2011), however, it cannot be excluded that other inhibitors impaired hydrolysate-based NEtGly production. Unlike *E. coli* and yeasts, *C. glutamicum* can simultaneously utilize acetate present in hydrolysates with other carbon sources (Wendisch et al., 2000). Pentose utilization that is slower than glucose utilization was accelerated by using alternative donors for enzymes of xylose catabolism and/or by pentose uptake engineering (Sasaki et al., 2009; Meiswinkel et al., 2013; Brüsseler et al., 2018). Uptake of acetate by MctC (Jolkver et al., 2009) or methylammonium (and likely also ethylammonium) uptake by Amt (Meier-Wagner et al., 2001) have not yet been targeted to improve production based on these substrates. Currently, it is unknown how NEtGly is exported from the *C. glutamicum* cell. While some export due to diffusion is possible, it is likely that an export system accepting NEtGly is encoded in the *C. glutamicum* genome. While transport engineering is expected to affect volumetric productivity, improvement of yield and titer requires other approaches of strain and process development.

In this study, we established fermentative production of the *N*-alkylated amino acid NEtGly. Expression of the *N*-methylamino acid dehydrogenase/IRED gene *dpkA* from *P. putida* in a recombinant *C. glutamicum* strain allowed reductive amination of glyoxylate forming NEtGly, when MEA was added. However, the xylose-based process had a low volumetric productivity (0.04 g L^−1^ h^−1^). By site directed mutagenesis of the substrate binding site, we could identify a mutant showing increased catalytic efficiency toward ethylamination of glyoxylate. Implementation of the mutant DpkA^F117L^ enabled faster production of NEtGly with a final titer of 6.1 ± 0.3 g L^−1^ and a volumetric productivity of 0.11 g L^−1^ h^−1^.

## Data Availability Statement

All datasets generated for this study are included in the manuscript/Supplementary Files.

## Author Contributions

MM and VW planned and designed the experiments. MM and MH performed the enzymatic characterization. MM constructed production strains and performed shake flask cultivations. SH and KS prepared lignocellulosic hydrolysates and SH and JR performed bioreactor cultivations. MM, MH, SH, JR, KN, and VW analyzed the data. MM drafted the manuscript. VW and KN finalized the manuscript. All authors agreed to the final version of the manuscript.

### Conflict of Interest

The authors declare that the research was conducted in the absence of any commercial or financial relationships that could be construed as a potential conflict of interest.
