# Supplementary material for: Fermentative Production of N-Alkylated Glycine Derivatives by Recombinant Corynebacterium glutamicum Using a Mutant of Imine Reductase DpkA From Pseudomonas putida
Source: Front Bioeng Biotechnol. 2019 Sep 26;7:232. doi: 10.3389/fbioe.2019.00232 (PMC6775277; doi:10.3389/fbioe.2019.00232)
Supplement: Supplementary file 1 [file Data_Sheet_1.PDF]

Supplementary Material

1 Supplementary Figures

A

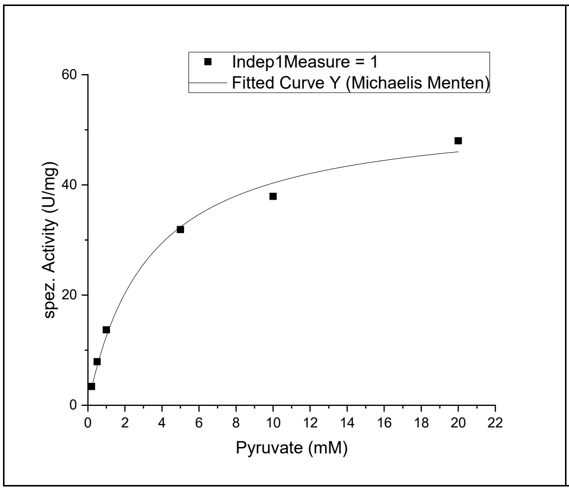

B

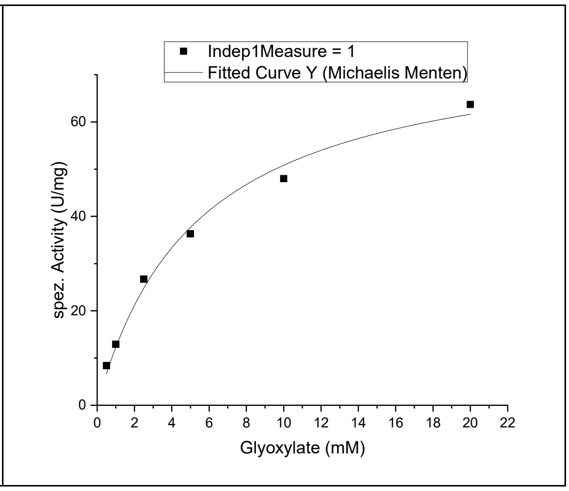

C

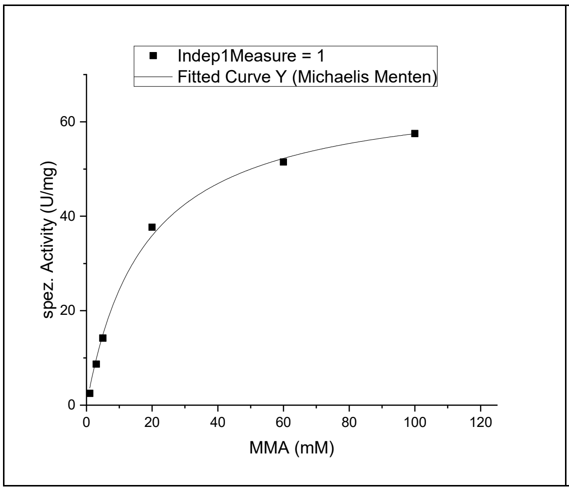

D

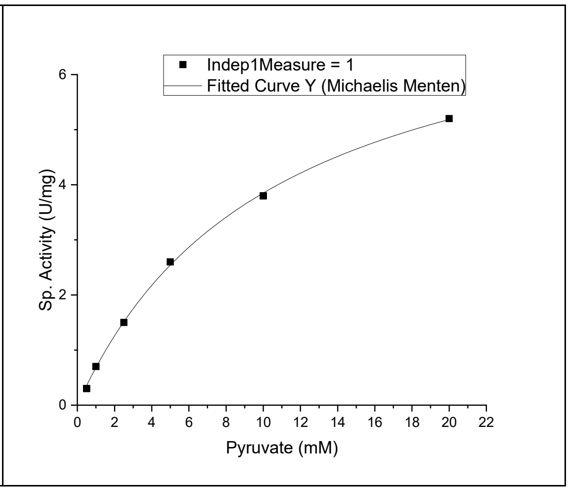

E

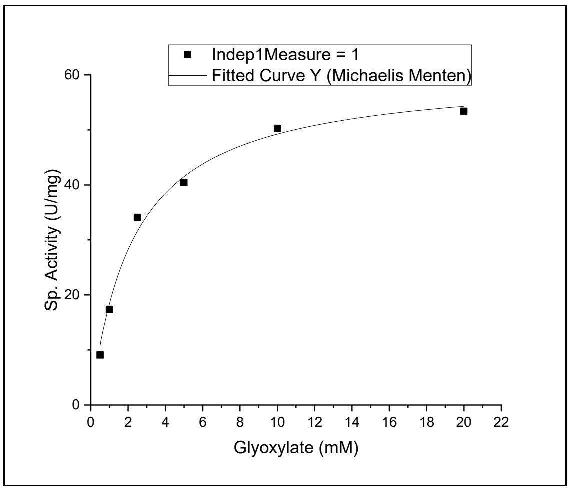

Supplementary Figure S1: Michaelis-Menten kinetics for DpkA wildtype enzyme using different substrates. Determination of K_m for pyruvate with similar MMA concentration (A), for glyoxylate with similar MMA concentration (B), for MMA with similar pyruvate concentration (C), for pyruvate with similar MEA concentration (D) and for glyoxylate with similar MEA concentration (E). Determination of K_m values were performed using Origin with the add-on 'Enzyme kinetics', k_{cat} calculations were performed with respect to Michaelis and Menten (translation: Johnson and Goody, 2011). The values are given in Table 2.

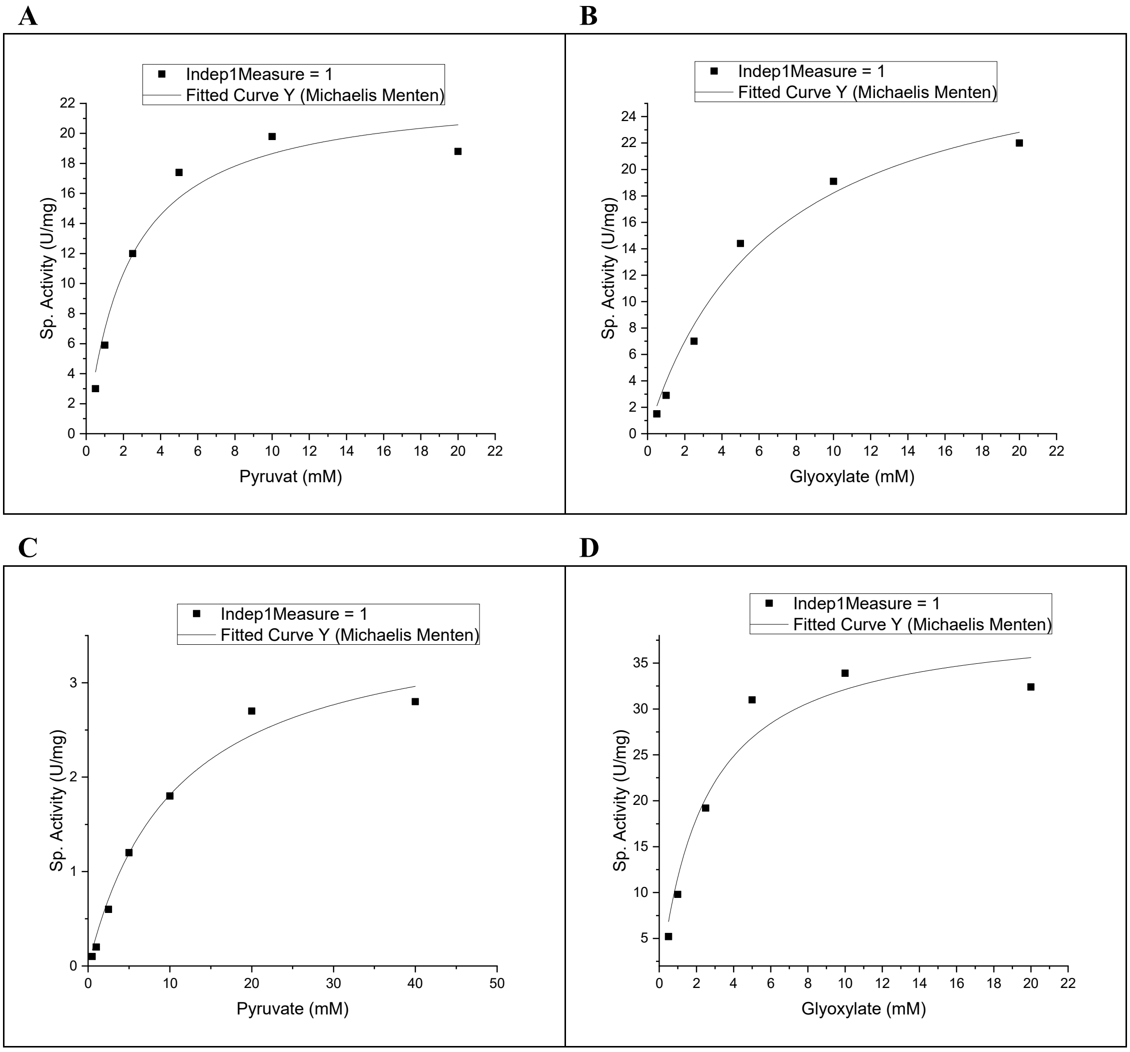

Supplementary Figure S2: Michaelis-Menten kinetics for DpkA^{F117L} enzyme using different substrates. Determination of K_m for pyruvate with similar MMA concentration (A), for glyoxylate with similar MMA concentration (B), for pyruvate with similar MEA concentration (C) and for glyoxylate with similar MAA concentration (D). Determination of K_m values were performed using Origin with the add-on ‘Enzyme kinetics', k_{cat} calculations were performed with respect to Michaelis and Menten (translation: Johnson and Goody, 2011).The values are given in Table 2.
